# Insights into Oxygen Migration in LaBaCo_2_O_6−δ_ Perovskites from *In Situ* Neutron Powder Diffraction and Bond Valence Site Energy Calculations

**DOI:** 10.1021/acs.chemmater.1c03726

**Published:** 2022-01-27

**Authors:** Fabian Hesse, Ivan da Silva, Jan-Willem G. Bos

**Affiliations:** † Institute of Chemical Sciences, Centre for Advanced Energy Storage and Recovery, School of Engineering and Physical Sciences, Heriot-Watt University, Edinburgh EH14 4AS, U.K.; ‡ ISIS Facility, Rutherford Appleton Laboratory, Harwell Oxford, Didcot OX11 0QX, U.K.

## Abstract

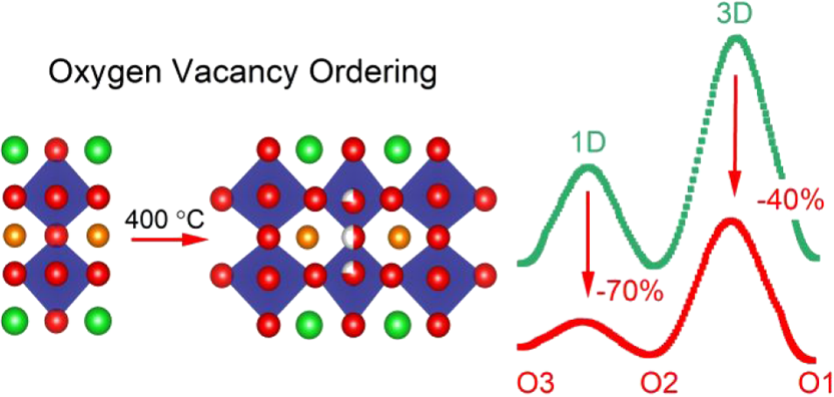

Layered cobalt oxide perovskites are important mixed ionic and electronic conductors. Here, we investigate LaBaCo_2_O_6−δ_ using *in situ* neutron powder diffraction. This composition is unique because it can be prepared in cubic, layered, and vacancy-ordered forms. Thermogravimetric analysis and diffraction reveal that layered and disordered samples have near-identical oxygen cycling capacities. Migration barriers for oxide ion conduction calculated using the bond valence site energy approach vary from *E*_b_ ∼ 2.8 eV for the cubic perovskite to *E*_b_ ∼ 1.5 eV for 2D transport in the layered system. Vacancy-ordered superstructures were observed at low temperatures, 350–400 °C for δ = 0.25 and δ = 0.5. The vacancy ordering at δ = 0.5 is different from the widely reported structure and involves oxygen sites in both CoO_2_ and LaO planes. Vacancy ordering leads to the emergence of additional migration pathways with low-energy barriers, for example, 1D channels with *E*_b_ = 0.5 eV and 3D channels with *E*_b_ = 2.2 eV. The emergence of these channels is caused by the strong orthorhombic distortion of the crystal structure. These results demonstrate that there is potential scope to manipulate ionic transport in vacancy-ordered LnBaCo_2_O_6−δ_ perovskites with reduced symmetry.

## Introduction

Dimensionality is an important design concept in solid-state chemistry with functional properties intimately linked to features in the crystal structure, for example, the empirical link between high-temperature superconductivity and layered structures and low-energy migration pathways in superionic materials.^1−3^ Layered cobalt double perovskites, LnBaCo_2_O_6−δ_ (Ln = lanthanides, Y), have been identified as promising electrode materials for solid oxide fuel cells^4−11^ as electrocatalysts for oxygen evolution in alkaline solution^12,13^ and also have interesting magnetic and thermoelectric properties.^14−16^ The strong performance in electrochemical applications is linked to their high electronic and ionic conduction, high surface oxygen exchange rates, and catalytic selectivity.^8,9,17−19^

**Figure 1:**
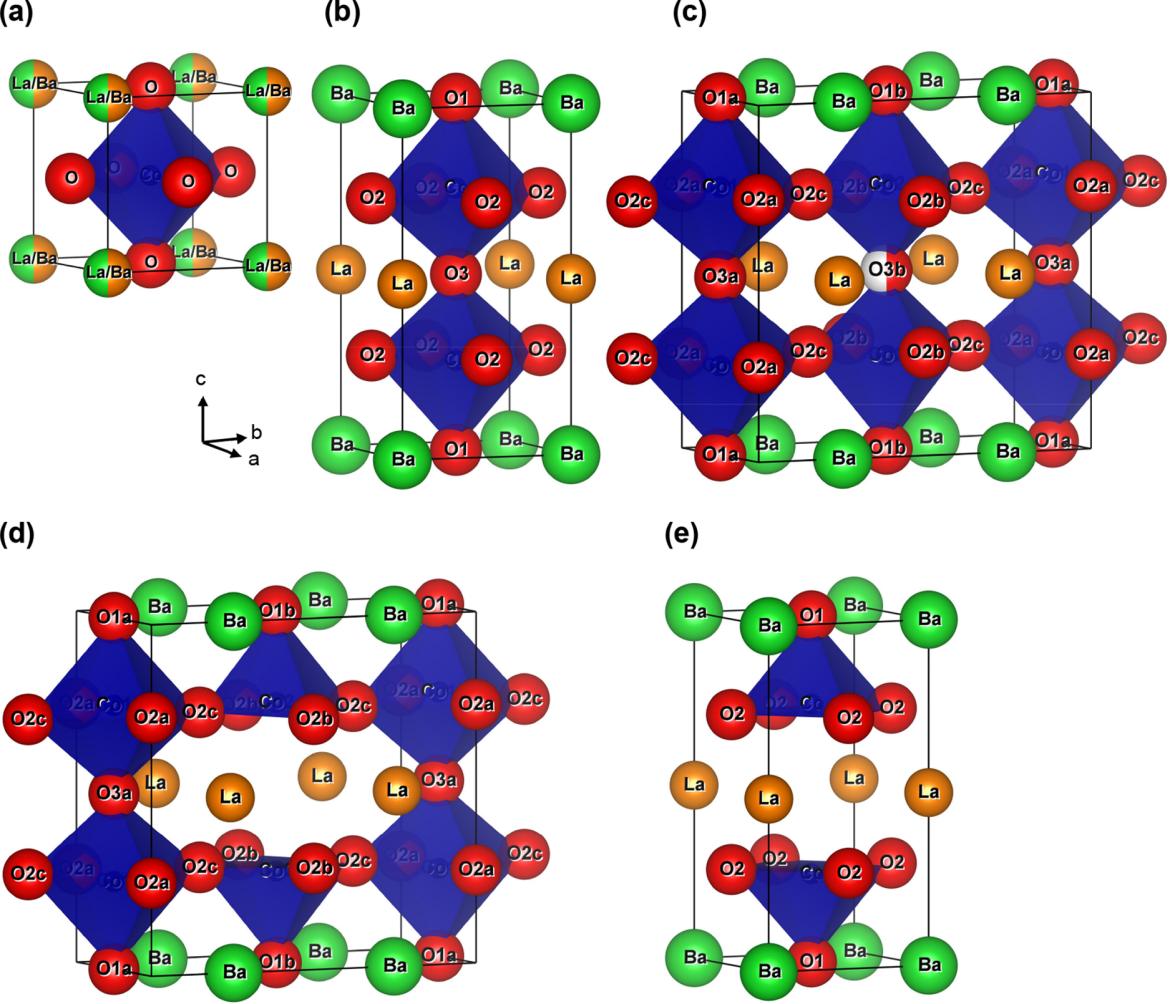
Schematic representation of the unit cell of: (a) cubic (*Pm*3̅*m*) La_0.5_Ba_0.5_CoO_3−δ_; (b) tetragonal (*P*4/mmm) LaBaCo_2_O_6_; (c) orthorhombic (*Pmmm*) LaBaCo_2_O_5.75_ with a vacancy-ordered half-filled O3b site; (d) orthorhombic (*Pmmm*) LaBaCo_2_O_5.5_ with a vacant O3b site and (e) tetragonal (*P*4/mmm) LaBaCo_2_O_5_ with an empty O3 site. La, Ba, Co, and O are represented by orange, green, blue, and red spheres, respectively.

An overview of the structures relevant to this article is given in Figure 1.^8^ A cubic perovskite, Ln_0.5_Ba_0.5_CoO_3−δ_, with disordered A-site cations and a lattice parameter *a*_p_ is shown in Figure 1a. Fully oxygenated LnBaCo_2_O_6_ (δ = 0, Co^3.5+^) has a similar perovskite network of corner-sharing CoO_6_ octahedra but with Ln and Ba in alternating layers (Figure 1b). This leads to a unit cell with *a*_p_ × *a*_p_ × 2*a*_p_ dimensions. Fully reduced LnBaCo_2_O_5_ (δ = 1, Co^2.5+^) has only square pyramidal CoO_5_ polyhedra and a deoxygenated LnO layer (Figure 1e). At intermediate δ values, a range of more complex structures, characterized by oxygen vacancy ordering, are observed.^8,20,21^ Two vacancy-ordered structures that are relevant to this work are shown in Figure 1c,d. The first is stable near δ = 0.25 (Co^3.25+^) and has a 50% filled O3b site in the LnO layer, leading to an orthorhombic structure with *a*_p_ × 2*a*_p_ × 2*a*_p_ unit cell. The second occurs near δ = 0.5 (Co^3+^) and has an alternating arrangement of square pyramidal CoO_5_ and octahedral CoO_6_ polyhedra. This structure has the same orthorhombic unit cell with a fully depopulated O3b site. Both structures are characterized by 1-dimensional (1D) vacancy-rich channels running parallel to the crystallographic *a*-direction. At higher vacancy fractions (δ = 5/9; 4Co^3+^ and 5Co^2+^), a structure with 3*a*_p_ × 3*a*_p_ × 2*a*_p_ tetragonal cell is observed for Ln = Y, Dy.^22,23^ This structure is characterized by two perpendicular 1D vacancy channels. In general, the vacancy-ordered structures are observed at or near room temperature, but they can be stable at elevated temperatures depending on Ln and oxygen partial pressure (*p*_O_2__).^24,25^

High-temperature *in situ* studies under varying *p*_O_2__ tend to use X-ray powder diffraction, which allows changes in the lattice metrics to be monitored. However, it does not allow accurate determination of oxygen site occupancies due to the weak scattering from oxygen. Neutron powder diffraction (NPD) is more suited due to the stronger scattering from the oxygen nucleus. Several *in situ* NPD studies have been reported, including work by Cox-Galhotra *et al.* who investigated LnBaCo_2_O_6−δ_ (Ln = Pr, Nd) between 577 and 852 °C and in 10^–1^ to 10^–4^ atm oxygen.^26,27^ This showed gradual removal of oxygen from O3 and O2 sites (Figure 1b) under reducing conditions (0.5 ≤ δ ≤ 0.9), but no oxygen vacancy-ordered superstructures were observed. Similarly, Garcés *et al.* use NPD in their study, contrasting cubic and layered LaBaCo_2_O_6−δ_ (up to 300 °C) but do not observe superstructures.^28^

Oxygen migration in LnBaCo_2_O_6−δ_ materials has attracted significant interest, both experimentally and from calculations, and proceeds *via* a vacancy-hopping mechanism.^7,8^ Here, the hopping frequency and ionic diffusion are largely controlled by the migration barrier *E*_b_.^29^ Molecular dynamics (MD) and density functional theory (DFT) calculations typically focus on the LnBaCo_2_O_5.5_ (δ = 0.5) composition (often with Ln = Pr and Gd, which are considered the best-performing compositions). Two somewhat contrasting mechanisms for ionic conduction have emerged from this work. One considers the O3 site as a vacancy reservoir with the lowest *E*_b_ for direct O2–O2 jumps, that is, ionic transport occurs predominantly within the CoO_2_ planes (Figure 1b).^30−32^ The other has the lowest *E*_b_ between O2–O3 sites, leading to an O2–O3–O2 migration path involving LnO and adjacent CoO_2_ planes.^33−35^ In both cases, the transport is essentially 2D with a large penalty for traversing the Ba–O layer. Maximum entropy analysis of residual scattering (at high temperature in the tetragonal phase) supports the 2D nature of ionic conduction involving both O2 and O3 sites.^36,37^ Furthermore, MD simulations show that despite the lower *E*_b_ of the O2–O2 path, the occurrence of O2–O3 jumps is higher, presumably due to the larger vacancy concentration on the O3 site.^31^ Typical *E*_b_ values are 0.3–0.8 eV from MD,^31,33,34^ while DFT studies yield similar 0.4–1 eV for low-energy migration paths.^32,35^ There is no evidence for direct hopping between vacancy-rich O3 sites, either in the tetragonal or vacancy-ordered orthorhombic structures, consistent with the large distance between O3 sites (∼3.8 Å).

The impact of cation ordering has been probed using MD simulations. This reveals a ∼67% decrease in the oxygen diffusivity on disordering Pr/Ba in PrBaCo_2_O_5.5_,^38^ but interestingly, the calculated *E*_b_ remains similar at ∼0.6 eV.^39^

Experimental *E*_b_ values from isotope exchange depth profile experiments are 0.5–1 eV (Ln = Pr) and 0.6 eV (Ln = Gd) and are in line with the results of simulations.^4,40^ Electrochemical impedance spectroscopy (EIS) measurements on symmetric cells, where electrical conduction is blocked by an insulating oxide ion conductor, yield larger activation energies *E*_a_ = 1.4–0.9 eV (Ln = La–Y) with a large scatter in the reported values.^9^ These *E*_a_ are determined from fitting high-frequency arcs in EIS, which are considered to be dominated by oxygen bulk and surface diffusion but are also affected by microstructure. It remains challenging to directly measure ionic conductivity in these systems due to the large electrical conductivity, for example, σ_e_ = 500–1000 S cm^–1^ at 200 °C for layered and disordered LaBaCo_2_O_6−δ_.^24^

Recently, bond valence site energy (BVSE) calculations have emerged as a computationally inexpensive route to calculate ionic migration pathways.^41,42^ BVSE is a force field approach where the energy of a “tracer” ion is calculated on a fine grid within the unit cell. The energy is described using a Morse-type interatomic potential, containing both attractive and short-range Born repulsions, and a Coulomb term for next-nearest-neighbor repulsions. The uniqueness of the BVSE approach is that the Morse potential is defined in terms of the bond valence parameters *R*_0_ and *b* which have traditionally been used to describe the empirical relationship between bond valence and bond distance. These are well-established and have been tabulated for a wide range of systems.^43−45^ BVSE therefore affords a comprehensive and computationally cheap way to determine energy surfaces for ionic migration.^41^ This contrasts with DFT, where calculations are generally undertaken only in pre-selected directions due to the higher computational cost. MD is another force field approach, but it calculates ionic diffusivities at high (simulation) temperatures and extracts migration barriers from the Arrhenius temperature dependence. By contrast, BVSE uses fixed atomic positions and does not consider thermal motion. BVSE has been applied to a wide range of systems and has been found to give good estimates for trends in migration barriers.^41,42,46−49^ Like other modeling approaches, the absolute values are only a proxy and the main strength of BVSE is in establishing relative migration barriers for related structures, such as the work described here.

Here, we focus on the LaBaCo_2_O_6−δ_ system, which can be prepared in a disordered cubic perovskite (La_0.5_Ba_0.5_CoO_3−δ_) and layered form and is stable over a wide range of oxygen stoichiometries.^24,25,28,50−52^ Both forms were probed using thermogravimetric analysis (TGA) and *in situ* NPD under flowing N_2_ between RT and 1000 °C. Vacancy-ordered superstructures are observed in the layered system for δ = 0.25 and δ = 0.5 at 350–400 °C. The latter involves vacancy ordering on two oxygen sites (both O2 and O3) that is different from that of the structure in Figure 1d. We report on the evolution of oxygen site occupancies and other structural parameters for both structure types and use BVSE calculations to provide a detailed comparison of oxygen ion migration. This confirms that the large Ba^2+^ ion prevents oxygen migration and reveals that structural distortions linked to the vacancy ordering cause low-energy pathways for ionic migration. This suggests that oxygen vacancy ordering may enable improved ionic conductivity at moderate temperatures.

## Experimental Section

### Synthesis

10 g of polycrystalline La_0.5_Ba_0.5_CoO_3−δ_ and LaBaCo_2_O_6−δ_ samples were prepared by solid-state reaction. Stoichiometric amounts of La_2_O_3_ (Sigma-Aldrich, 99.999%), Co_3_O_4_ (Alfa Aesar, 99.9985%), and BaCO_3_ (Alfa Aesar, 99.997%) were mixed using a mortar and pestle and annealed in a muffle furnace for 12 h at 1000 °C. To obtain disordered La_0.5_Ba_0.5_CoO_3−δ_, pellets of the annealed mixture were sintered in air at 1100 °C for 12 h, with heating and cooling rates of 10 °C min^–1^. Ordered LaBaCo_2_O_6−δ_ was obtained by sintering cold-pressed pellets in a tube furnace at 1150 °C (2 °C min^–1^ heating and cooling) for 48 h under flowing Ar. All the characterization presented in this article was done on the same samples.

### Characterization

Initial phase analysis was undertaken using X-ray powder diffraction using a Bruker D8 Advance diffractometer with monochromated Cu Kα_1_ radiation. High-quality data sets were collected over 7 h. Iodometric titration was used to evaluate the Co oxidation state and determine the oxygen content after synthesis. 4 M HCl solution was saturated by bubbling Ar through the solution for a minimum of 30 min. 1 g of KI and ∼20 mg of perovskite oxide was dissolved in the HCl solution and titrated against 0.01 M Na_2_S_2_O_3_ solution under argon atmosphere. This procedure yielded the oxygen content δ = 0.00(6) for disordered La_0.5_Ba_0.5_CoO_3−δ_ and δ = 0.46(4) for layered LaBaCo_2_O_6−δ_, consistent with the synthesis in air and under Ar for these samples, and with literature reports.^24,28,52^ The reported error is the standard deviation of triplicate measurements. TGA data were collected under flowing N_2_ (BOC oxygen-free nitrogen, *p*_O_2__ ≈ 10^–5^ atm, 100 cm^3^ min^–1^) using a Linseis STA PT 1600 instrument. Measurements were done on ∼200 mg of the sample contained in an alumina crucible.

### Neutron Powder Diffraction

Time-of-flight NPD data were collected on ∼5 g of the powdered sample using the GEM diffractometer at the ISIS Neutron and Muon Source, Rutherford Appleton Laboratory, UK. The samples were loaded into a double-walled quartz gas-flow holder (Figure S1 in the Supporting Information) and heated between 25 and 1000 °C under N_2_ flow, using the same N_2_ gas and flow as used in the TGA experiment. Data were collected for ∼350 μA h proton beam current, corresponding to ∼2 h exposure at each temperature. Background measurements on an empty quartz holder were carried out at 25, 400, 700, and 1000 °C. These data sets were used to fix the background in the Rietveld analysis of the data sets collected on the La_0.5_Ba_0.5_CoO_3−δ_ and LaBaCo_2_O_6−δ_ samples. Rietveld analysis was carried out using GSAS II software.^53,54^ A small linear absorption correction (μ*R* = 0.2) was applied. The crystal structures were visualized using VESTA software.^55^

### Bond Valence Sum and Bond Valence Sum Energy Calculations

BVS calculations were used to determine the oxidation states of metal cations and oxygen anions.^44^ We determined the following BVS parameters for high-spin Co^3+^: *R*_0_ = 1.74 and *b* = 0.37. These values yield excellent agreement with the Co oxidation state from Rietveld analysis between RT–1000 °C and the titration experiments for both samples (Tables 1 and 3). Hence, there is agreement between the Co oxidation state obtained from bond distances and site occupancy analysis. For the higher temperature data sets, the BVS parameters were corrected for thermal expansion (α ≈ 2.0 × 10^–5^ K^–1^),^56^ which was obtained from the NPD data as outlined in the Supporting Information (Table S1 and Figure S2). The obtained α is in good agreement with literature data.^8,9^ BVSE calculations were undertaken using SoftBV software,^41^ with the BVS parameter file updated to reflect the values determined for high-spin Co^3+^. Temperature-corrected BVS parameters were used in the BVSE calculations, although the impact on the calculated *E*_b_ is small (<0.2 eV). BVSE maps are calculated with a resolution of 0.01 Å and plotted as constant energy isosurfaces or as plots of energy versus reaction coordinates for low-energy oxygen migration paths.

**Table 1:** Fitted Lattice Parameter, Oxygen Content Obtained from the Oxygen Site Occupancy (Ox. Content), Co Oxidation State from Fitted Chemical Composition (Co^*x*+^), Bond Valence Sum (BVS) for Co and O, and Goodness of Fit (*wR*_P_) for La_0.5_Ba_0.5_CoO_3−δ_ Fitted against NPD Data between RT and 1000 °C upon Heating and at 300 °C and RT on Cooling^a^

	RT	250 °C	400 °C	550 °C	700 °C	850 °C	1000 °C	300 °C–*c*	RT °C–*c*
*a* (Å)	3.8917(1)	3.9104(1)	3.9287(1)	3.9501(2)	3.9734(2)	3.9919(2)	4.0130(2)	3.9372(3)	3.9206(3)
Ox. cont.	2.98(3)	3.00(3)	2.92(3)	2.84(3)	2.78(3)	2.72(3)	2.60(3)	2.72(2)	2.70(2)
Co^*x*+^	3.46(6)	3.50(6)	3.34(6)	3.18(6)	3.06(6)	2.93(6)	2.71(6)	2.95(6)	2.91(6)
BVS (Co)	3.42(5)	3.43(6)	3.30(7)	3.16(8)	3.04(9)	2.9(1)	2.8(1)	3.01(6)	2.98(5)
BVS (O)	2.10(5)	2.08(6)	2.05(7)	2.02(8)	1.98(9)	2.0(1)	1.9(1)	2.01(6)	2.00(5)
*wR*_P_ (%)	2.05	1.74	1.43	1.45	1.43	1.37	1.35	2.21	2.33

a Space group: *Pm*3̅ *m*; La 1a (0 0 0); Ba 1a (0 0 0); Co 1b (0.5 0.5 0.5); and O 3c (0.5 0.5 0). ADPs are given in Table S3.

## Results and Discussion

### Thermogravimetric Analysis

**Figure 2:**
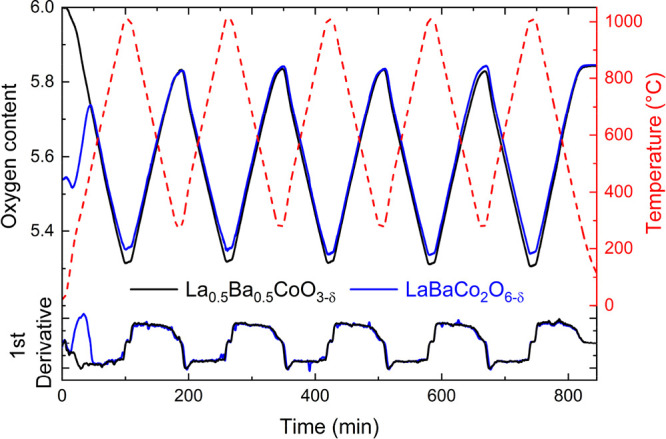
TGA of disordered La_0.5_Ba_0.5_CoO_3−δ_ and layered LaBaCo_2_O_6−δ_ over five heat–cool cycles (RT–1000 °C, then 4 × 300-1000 °C) under N_2_ flow. The first derivate illustrates the near-identical response to changing temperature, demonstrating that there is no difference in oxygen uptake/loss between these materials on the timescale of the TGA measurement. Both materials have very similar cycling capacity in the range of 0.2 < δ < 0.7. The oxygen content of La_0.5_Ba_0.5_CoO_3−δ_ was doubled for ease of comparison.

Figure 2 shows the change in oxygen content for La_0.5_Ba_0.5_CoO_3−δ_ and LaBaCo_2_O_6−δ_ over five heat–cool cycles (RT–1000 °C, then 4 × 300–1000 °C, 10 C min^–1^ heating and cooling). Both samples follow the temperature profile in producing oxygen vacancies during heating and gaining oxygen during cooling. During the first heating step, LaBaCo_2_O_6−δ_ (δ ∼ 0.5 after synthesis) gained weight by absorbing oxygen. After this step, the measured oxygen capacities of both samples showed near-identical performances with a reversible oxygen loss and gain of ∼0.52 mol O (3.4 wt %); (0.2 ≤ δ ≤ 0.7) featuring high stability and durability through five heat–cool cycles. The first derivatives of the oxygen loss are nearly identical (Figure 2), confirming the similar uptake and oxygen loss characteristics of the two samples. The lattice parameters before and after cycling are unchanged (Table S2), consistent with the reversible loss and uptake of oxygen. Stepwise heating of La_0.5_Ba_0.5_CoO_3−δ_ between 100 and 1000 °C (Figure S3) shows that the oxygen content follows temperature (*p*_O_2__) instantaneously. This confirms that the kinetics to equilibrate is faster than the timescale of the measurement and that the TGA measurements probe the underlying thermodynamic stability, which appears dictated by the Co oxidation state and not by details of the crystal structure.

### NPD of Disordered La_0.5_Ba_0.5_CoO_3−δ_

**Figure 3:**
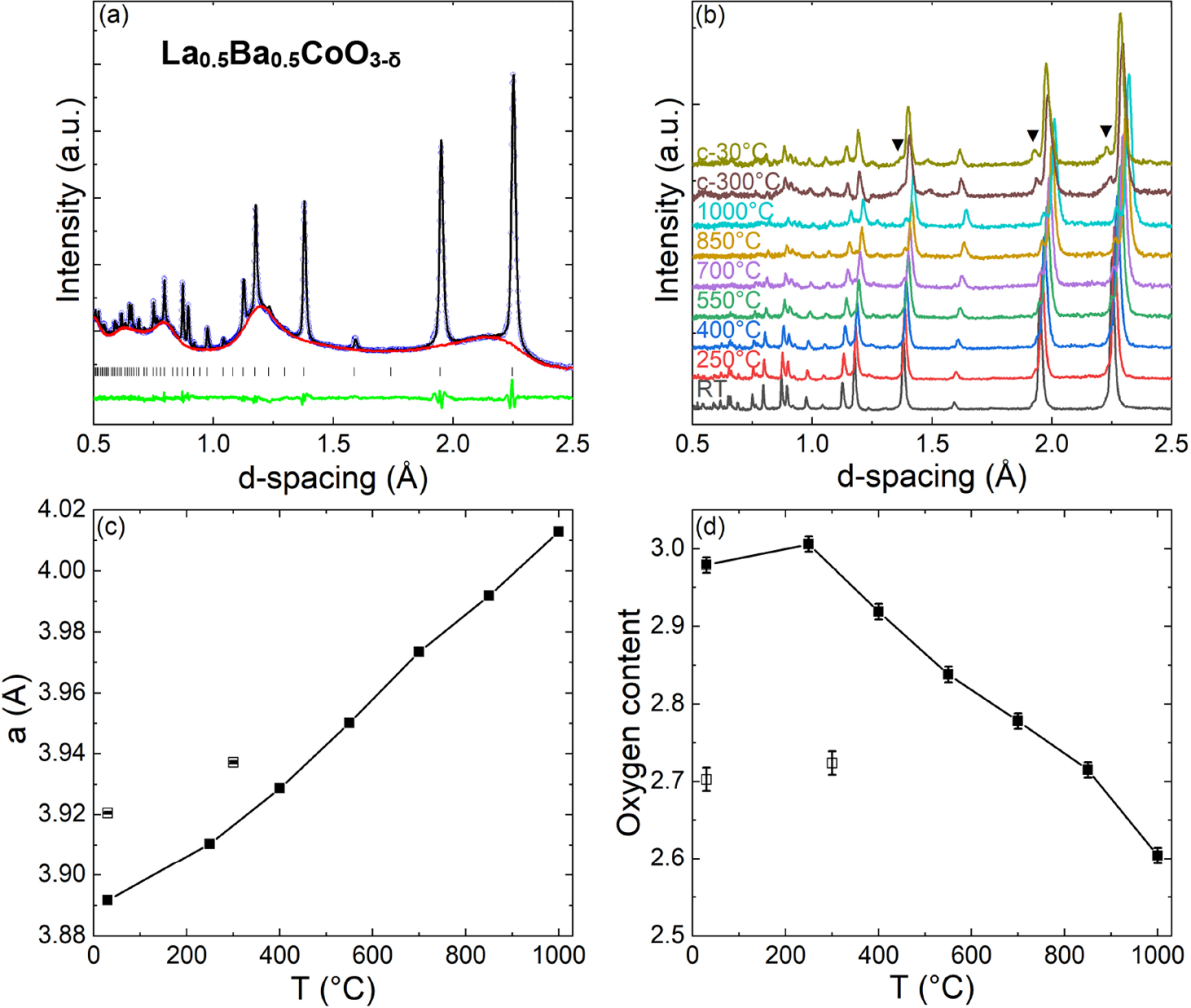
(a) Rietveld fit (black line) to GEM NPD data (open circles) collected on disordered La_0.5_Ba_0.5_CoO_3−δ_ at RT under N_2_ flow. The red line is the fitted background from an empty sample holder, the green line is the difference curve, and the peak positions are indicated by vertical markers. (b) Stacked La_0.5_Ba_0.5_CoO_3−δ_ NPD patterns upon heating from RT to 1000 °C and after cooling under N_2_ flow. ▼ represents the La_2_O_3_ impurity that emerges at high temperatures. The two remaining panels show the temperature dependence of (c) the lattice parameter and (d) the overall oxygen content. Data on cooling are shown as open symbols.

Rietveld analysis confirmed the presence of the cubic perovskite structure with a = 3.8922(1) Å and δ = 0.02(3) after synthesis (Figure 3a, Table 1). Variable temperature NPD patterns (Figure 3b, RT–1000 °C, then 350 °C, and RT on cooling) are consistent with the coupled effects of thermal expansion and chemical reduction and confirm the absence of phase changes. However, an increasing amount of La_2_O_3_ was detected above 500 °C (Figure S4), suggesting that prolonged exposure to high temperatures is causing a slow decomposition of La_0.5_Ba_0.5_CoO_3−δ_. This impurity phase was not evident after the TGA experiments due to the much shorter exposure to high temperatures. Refinement of the La/Ba ratio shows that this remains at 1:1, suggesting that La_0.5_Ba_0.5_CoO_3−δ_ decomposes rather than selectively losing La in the form of La_2_O_3_. The absence of other impurity phases suggests that the decomposition could be due to the sublimation of a Ba–Co–O phase. During heating, La_0.5_Ba_0.5_CoO_3−δ_ linearly releases oxygen from ∼250 °C until reaching δ = 0.40(1) at 1000 °C (Figure 3c,d), in agreement with the TGA results. Anisotropic oxygen atomic displacement parameters (ADPs) show a strong motion perpendicular to the Co–O–Co bonds (Table S3 and Figures S5a and S7a), consistent with the curved path expected for ionic migration (also the BVSE section below). Unlike in the TGA experiment, the structure does not return to the fully oxidized (δ = 0) state on cooling under flowing N_2_. This discrepancy is likely caused by a tighter vacuum in the NPD sample environment and is also observed for the layered LaBaCo_2_O_6−δ_ sample.

### NPD of Ordered LaBaCo_2_O_6−δ_

**Figure 4:**
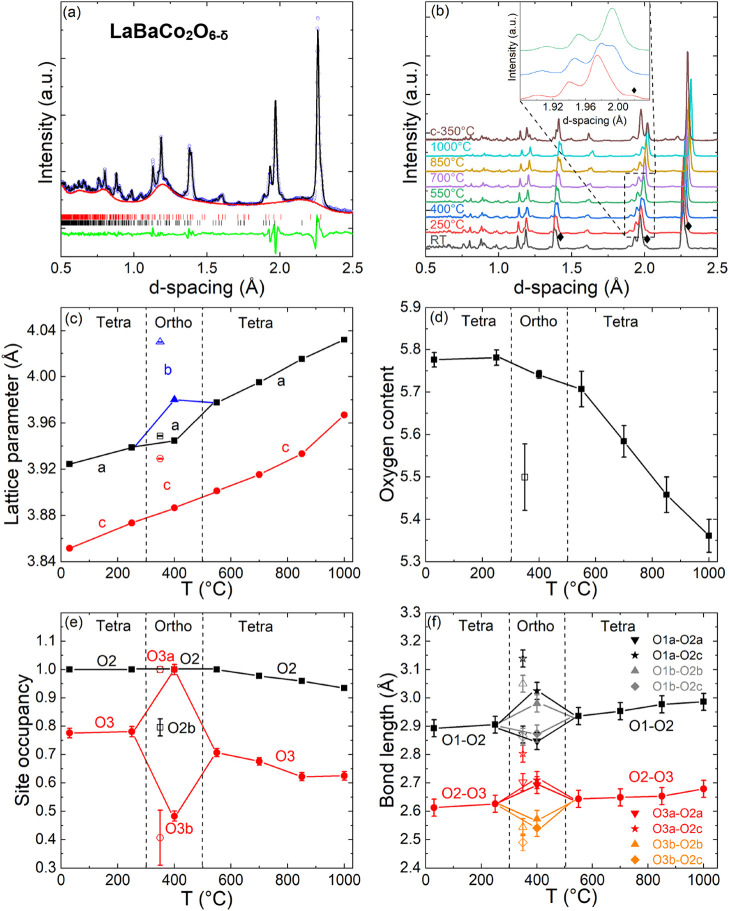
(a) Rietveld fit (black line) to GEM NPD data (open circles) collected on ordered LaBaCo_2_O_6−δ_ at RT under N_2_ flow. The red line is the fitted background from an empty sample holder, the green line is the difference curve, and the two LaBaCo_2_O_6−δ_ phases are indicated by black (δ ∼ 0.2) and red vertical (δ ∼ 0.5) markers. (b) Stacked LaBaCo_2_O_6−δ_ NPD patterns upon heating from RT to 1000 °C and after cooling under N_2_ flow. The inset illustrates the peak splitting at ∼2.0 Å indicative of the orthorhombic superstructure. ⧫ in the RT pattern represents the δ = 0.5 phase, which merges with the main LaBaCo_2_O_6−δ_ phase on heating. The remaining panels show the temperature dependence of the: (c) lattice parameters; (d) overall oxygen content; (e) oxygen site occupancies; and (f) O–O distances. Data on cooling are shown as open symbols.

The quality of the Rietveld fit of the RT pattern is illustrated in Figure 4a. The variable temperature NPD data reveal the presence of orthorhombic superstructures on heating at 400 °C and on cooling at 350 °C (Figure 4b), while the structure is tetragonal at all other temperatures. All fitted structural parameters are summarized in Table 2, while anisotropic ADPs are given in Table S4. The evolution of the unit cell parameters, oxygen content, and site occupancies is given in Figure 4c–e, respectively, with O–O bond distances given in Figure 4f.

**Table 2:** Fitted Lattice Parameters, Unit Cell Volume, Fractional Coordinates and Site Occupancies, Oxygen Content Obtained from the Oxygen Site Occupancy (Ox. Content), Co Oxidation State from Fitted Chemical Composition (Co^*x*+^), and Goodness of Fit (*wR*_P_) Rietveld Fit Parameters for LaBaCo_2_O_6−δ_ between RT and 1000 °C upon Heating and at 350 °C after Cooling^a^

	RT–*a*	RT–*b*	250 °C–*a*	250 °C–*b*	400 °C	550 °C	700 °C	850 °C	1000 °C	350 °C–*c*
space group	*P*4/*mmm*	*P*4/*mmm*	*P*4/*mmm*	*P*4/*mmm*	*Pmmm*	*P*4/*mmm*	*P*4/*mmm*	*P*4/*mmm*	*P*4/*mmm*	*Pmmm*
*a* (Å)	3.9243(3)	3.910(5)	3.9388(3)	3.920(5)	3.9447(3)	3.9776(2)	3.9951(2)	4.0150(2)	4.0321(2)	3.9488(3)
*b* (Å)					7.9602(6)					8.0603(6)
*c* (Å)	7.7030(7)	8.02(1)	7.7471(7)	8.04(1)	7.7731(6)	7.8025(5)	7.8307(4)	7.8669(5)	7.9337(5)	7.8583(6)
*V* (Å^3^)	118.63(3)	122.6(1)	120.19(3)	123.5(1)	244.08(5)	123.45(2)	124.98(2)	126.82(2)	128.98(2)	250.11(5)
La (*y*)					0.241(1)					0.230(1)
Ba (*y*)					0.252(1)					0.238(1)
Co(tetr.)/Co1(orth.) (*z*)	0.256(1)	0.25(1)	0.256(1)	0.25(1)	0.249(1)	0.256(1)	0.254(1)	0.253(1)	0.251(1)	0.249(2)
Co2(orth.) (*z*)					0.260(2)					0.248(2)
O2(tetr.)/O2a(orth.) (*z*)	0.276(1)	0.27(1)	0.276(1)	0.27(1)	0.265(1)	0.277(1)	0.278(1)	0.279(1)	0.278(1)	0.265(1)
O2b(orth.) (*z*)					0.289(2)					0.296(2)
O2c(orth.) (*y*)					0.264(1)					0.275(1)
O2c(orth.) (*z*)					0.279(1)					0.282(1)
O2(tetr.)/O2b(orth.) Occ	1	1	1	1	1	1.00(2)	0.98(2)	0.96(2)	0.93(2)	0.80(3)
O3(tetr.)/O3b(orth.) Occ	0.78(2)	0.5(1)	0.78(2)	0.5(1)	0.48(2)	0.71(1)	0.68(1)	0.62(1)	0.63(2)	0.4(1)
Ox. cont.	5.78(2)	5.5(1)	5.78(2)	5.5(1)	5.74(1)	5.71(4)	5.58(4)	5.46(4)	5.36(4)	5.50(8)
Co^*x*+^	3.28(2)	3.0(1)	3.28(2)	3.0(1)	3.21(1)	3.21(4)	3.08(4)	2.96(4)	2.86(4)	3.00(8)
weight fraction (%)	90(1)	10(1)	91(1)	9(1)	100	100	100	100	100	100
*wR*_P_ (%)	4.11	3.82	2.43	3.06	2.54	2.41	2.06	2.57		

a *P*4/*mmm*: La 1b (0 0 0.5); Ba 1a (0 0 0); Co 2h (0.5 0.5 *z*); O1 1c (0.5 0.5 0); O2 4i (0.5 0 z); and O3 1d (0.5 0.5 0.5). *Pmmm*: La 2n (0 *y* 0.5); Ba 2m (0 *y* 0); Co1 2s (0.5 0 *z*); Co2 2t (0.5 0.5 *z*); O1a 1b (0.5 0 0); O1b 1f (0.5 0.5 0); O2a 2q (0 0 *z*); O2b 2r (0 0.5 *z*); O2c 4v (0.5 *y z*); O3a 1d (0.5 0 0.5); and O3b 1h (0.5 0.5 0.5). Oxygen site occupancies that are not listed (O1, O2a,c, and O3a) were refined to unity and kept fixed in the final fit cycles. ADPs are given in Table S4.

The RT data revealed the presence of an oxygen-rich [δ = 0.22(2)] main phase and an oxygen-poor [δ = 0.5(1)] minor phase (∼9:1 mixture, Table 2). The latter corresponds to the as-synthesized composition, revealing the majority of the ∼5 g sample oxidized during the 2–3 months of storage awaiting the NPD experiment. This was somewhat unexpected but is in keeping with the high-reported oxygen mobilities in these materials.^8,9^ There was no evidence for O vacancy ordering for the δ = 0.5 sample at RT, although the small weight fraction and peak overlap prohibit the unambiguous assignment of the structure. Iodometric titration of a stored sample confirmed the increased oxygen content with δ = 0.20(2), whereas the freshly prepared sample had δ = 0.46(4), both values in near-perfect agreement with the Rietveld analysis. The layered tetragonal structure has three oxygen sites: O1 is the apical oxygen site located in the Ba–O layer; O2 is the basal plane oxygen site; and O3 is the other apical oxygen site, located in the LaO layer (Figure 1b). In terms of redox properties: our fits reveal that O3 is depopulated first, followed by O2, while O1 remains fully occupied (Figure 4e). This is consistent with the literature results on the Ln = Pr and Nd systems.^26,27^ CoO_6_ octahedra are distorted with the basal plane O2 site displaced toward the LaO layer, leading to a ∼10% shorter O2–O3 distance (∼2.6 Å) compared to O1–O2 (∼2.9 Å). This distortion remains almost unchanged on heating (Figure 4f), showing that it is inherent to the La/Ba ordering and not affected by oxygen removal. The anisotropic ADP of the O2 site shows a large thermal motion perpendicular to the Co–O–Co bonds, as observed for La_0.5_Ba_0.5_CoO_3−δ_, consistent with a curved migration path between O2/O3 sites (Table S4, Figures S5 and S7). The O3 site has a similar large ADP, which coupled with the short distance between O2–O3 points toward the important role of these sites during oxygen migration. Similar ADP behavior was noted for the isostructural Ln = Pr and Nd systems that remain tetragonal throughout.^26,27^

**Figure 5:**
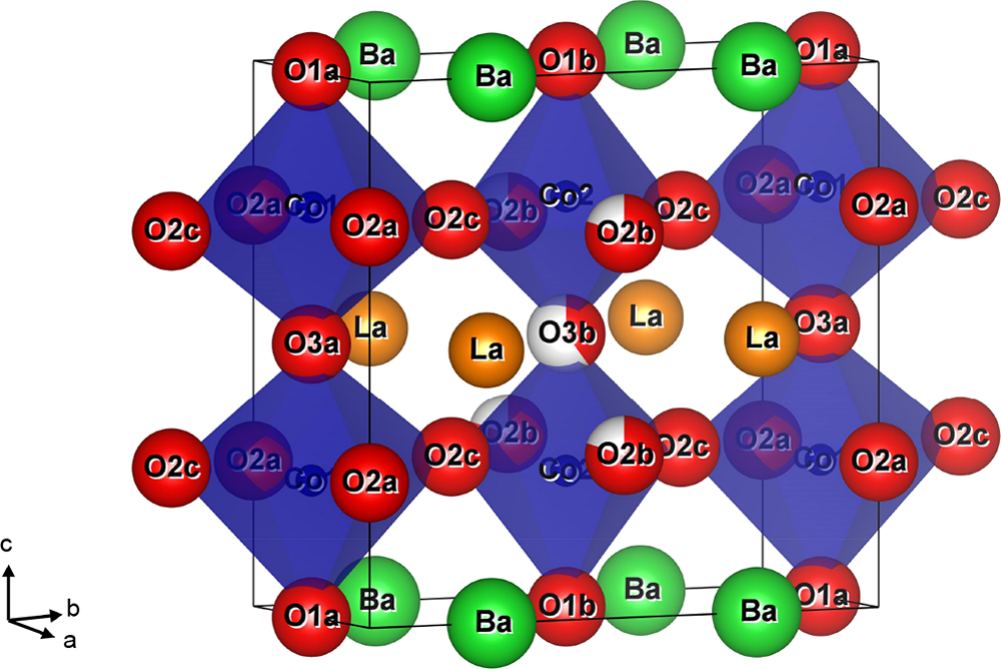
Schematic representation of the unit cell of orthorhombic (*Pmmm*) LaBaCo_2_O_5.50(8)_ with the vacancy-ordered ∼50% filled O3b site and ∼80% filled O2b site at 350 °C, after cooling. La, Ba, Co, and O are represented by green, orange, blue, and red spheres, respectively.

The orthorhombic superstructure on heating (400 °C, δ = 0.25) is characterized by a partial oxygen vacancy ordering in the LaO layer (Figure 1c). The O3 site splits into two positions, one of which is fully occupied (O3a) and the other has 50% occupancy (O3b). The lowered symmetry also leads to three different O2 positions. The consequence is a splitting of the O2–O3 connection into two short (∼2.55 Å) and two long (∼2.7 Å) distances (Figure 4f). The shortened distances are both to the 50% filled O3b site and form the low-energy migration paths, as discussed below. Upon heating, the vacancy ordering disappears and the basic layered tetragonal structure is observed up to 1000 °C. A second vacancy-ordered structure is observed at a similar temperature (350 °C) on cooling but with different overall oxygen content (δ = 0.5). The idealized ordering pattern for δ = 0.5 is alternating full/empty O3a/b sites, as illustrated in Figure 1d. However, this is not what is observed in our sample, and instead, a vacancy order involving both O3 and O2 sites occurs (Figure 5). Here, the O3b site is ∼50% filled (as for δ = 0.25) and the O2b site is ∼80% filled with all the other O sites fully occupied (Table 2 and Figure 4e). This structure also has two shortened (∼2.5 Å) and two elongated (∼2.7 and ∼2.8 Å) O2–O3 distances (Figure 4f), with the short distances linked to the 50% filled O3b site, identical to the δ = 0.25 structure. The anisotropic ADPs of the two vacancy-ordered superstructures are shown in Figure S7. The most significant change is the emergence of a large diagonal component for the O2c sites, pointing directly toward the O3b sites. This is not allowed in the basic tetragonal structure. The O2b sites maintain the typical large thermal motion perpendicular to the Co–O–Co bonds, reflective of a curved migration path.

The vacancy-ordered superstructures have two Co sites with strongly distorted octahedral coordination (Figure 5). The highly distorted nature makes the comparison of bond distances (Figure S6) difficult, and here, BVS calculations are useful to provide insights into the difference between the two Co sites. The calculated Co valences are: Co1: +3.04(7) and Co2: +3.31(7) for δ = 0.25 and Co1: +2.75(6) and Co2: +3.19(6) for δ = 0.5 (Table 3). Hence, the oxidation state of Co2 is higher and identical within two standard deviations, while Co1, which is not connected to the O2b/O3b vacancy sites, is selectively reduced. The size mismatch of the Co–O coordination polyhedra can be seen from the structural representation in Figure 5 and is a key factor in reducing steric hindrance, leading to the emergence of low-energy migration paths involving the O2b/O3b sites (discussed below). The average oxidation state is in good agreement with the expected values of +3.25 (δ = 0.25) and +3.00 (δ = 0.5). The BVS calculations demonstrate that a Co charge ordering occurs, coupled to the oxygen vacancy ordering, with the oxidation state difference of Δ*q* ∼ 0.25 (on heating) and Δ*q* ∼ 0.50 (on cooling). This charge ordering is different from the one that occurs in the 3*a*_p_ × 3*a*_p_ × 2*a*_p_ superstructure for Ln = Y, where Co^2+^ has square pyramidal and Co^3+^ has octahedral coordination, and the charge ordering is a direct consequence of the reduced coordination number.^22,23^ An opposite trend is observed here: the Co site with the highest coordination is the most reduced. This suggests that the charge ordering is not simply a consequence of the oxygen vacancy ordering, but occurs in parallel, and is at least part of the cause for the transition to the orthorhombic structure.

**Table 3:** Bond Valence Sums (BVSs) for LaBaCo_2_O_6−δ_ between RT and 1000 °C upon Heating and at 350 °C after Cooling

	RT	250 °C	400 °C	550 °C	700 °C	850 °C	1000 °C	350 °C–*c*
BVS(Co1)	3.23(5)	3.22(6)	3.04(7)	3.11(8)	3.01(9)	2.9(1)	2.8(1)	2.75(6)
BVS(Co2)			3.31(7)					3.19(6)
BVS(O1a)	2.10(5)	2.09(6)	2.18(7)	2.04(8)	2.06(9)	2.1(1)	2.1(1)	2.25(6)
BVS(O1b)			2.00(7)					1.99(6)
BVS(O2a)	2.06(5)	2.05(6)	2.08(7)	2.00(8)	1.98(9)	2.0(1)	1.9(1)	2.16(6)
BVS(O2b)			1.97(7)					1.69(6)
BVS(O2c)			2.05(7)					1.97(6)
BVS(O3a)	2.13(5)	2.12(6)	2.31(7)	2.08(8)	2.01(9)	2.0(1)	1.9(1)	2.36(6)
BVS(O3b)			2.36(7)					1.78(6)

### BVSE Analysis of Oxygen Migration Pathways

**Figure 6:**
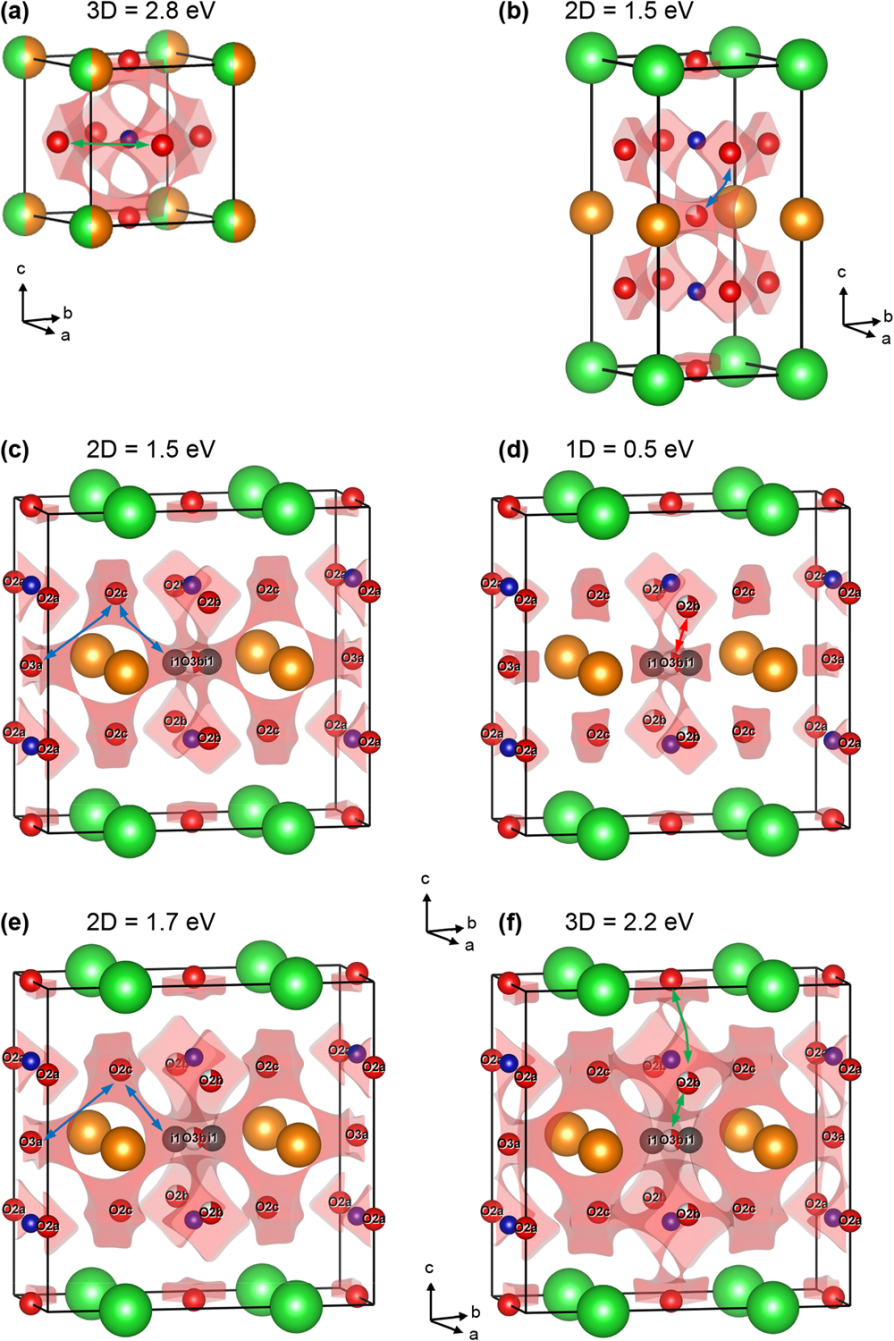
Energy isosurfaces from BVSE calculations for oxygen migration for (a) cubic La_0.5_Ba_0.5_CoO_3−δ_; (b) tetragonal LaBaCo_2_O_6−δ_; (c) orthorhombic LaBaCo_2_O_6−δ_ on heating at 400 °C (δ = 0.25); and (d–f) after cooling at 350 °C (δ = 0.5). The energies are set at the energy barrier (*E*_b_) enabling 1D, 2D, or 3D ionic transport. La, Ba, Co, and O are shown as orange, green, blue, and red spheres, respectively. The i1 split-site has similar coordinates (0.5, 0.44, 0.5) on both heating and cooling. The highest dimension migration paths are indicated using colored arrows that match the coding in Figure 7.

**Figure 7:**
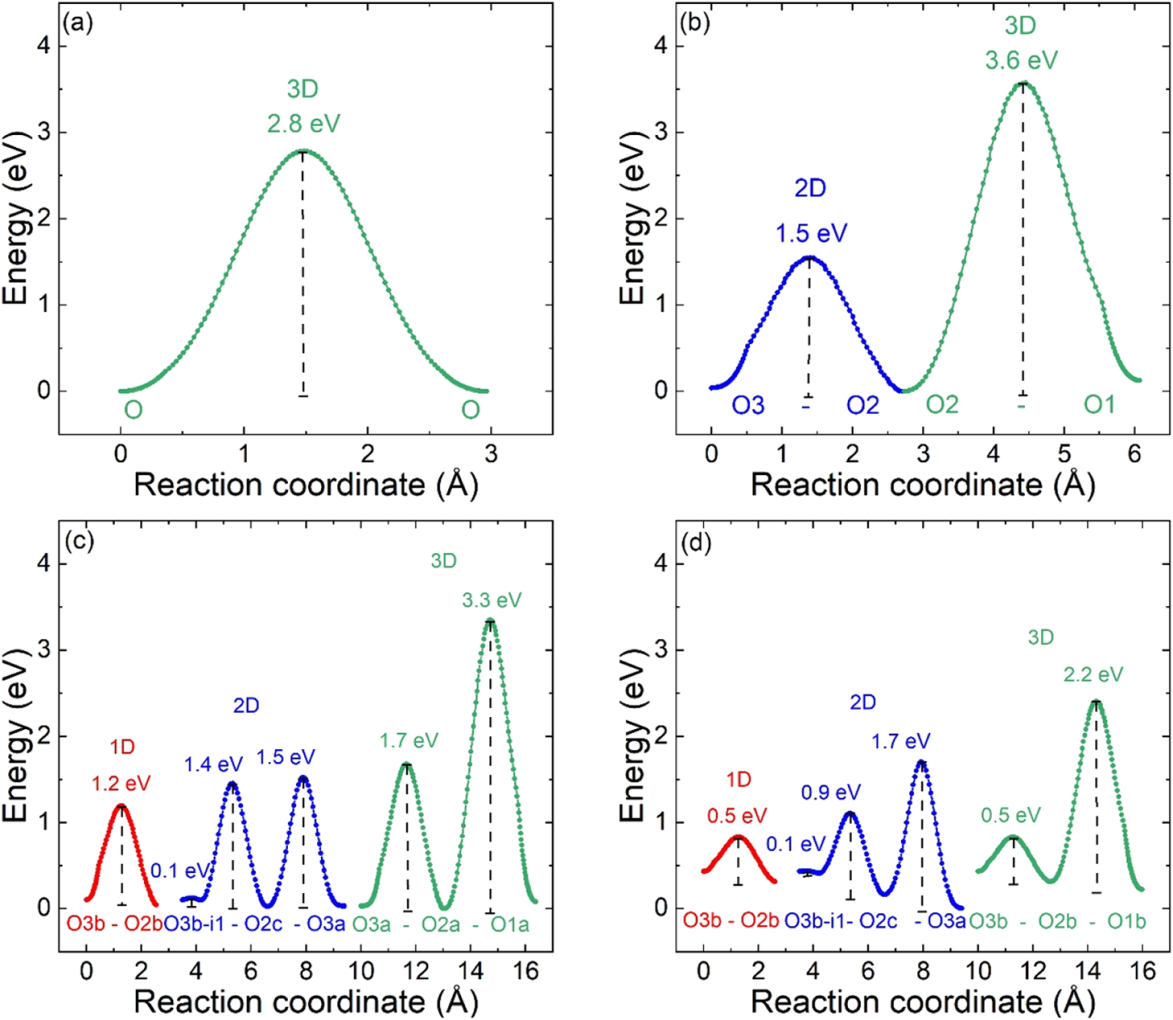
Activation barriers for oxygen migration from BVSE calculations for: (a) cubic La_0.5_Ba_0.5_CoO_3−δ_; (b) tetragonal LaBaCo_2_O_6−δ_; (c) orthorhombic LaBaCo_2_O_6−δ_ upon heating (400 °C, δ = 0.25); and (d) after cooling at 350 °C (δ = 0.5). RT structural data were used for the cubic and ideal tetragonal samples. The i1 position is a split-site adjacent to the vacancy-ordered O3b site with coordinates (0.5, 0.44, 0.5) and is separated by a small ∼0.1 eV energy barrier and ∼0.5 Å distance.

Constant energy isosurfaces at characteristic barrier energies (*E*_b_) and low-energy trajectories for ionic hopping are shown in Figures 6 and 7. The nodes in Figure 6 give the location of the barrier, that is the highest energy the ion will encounter during hopping between oxygen lattice sites. The cubic La_0.5_Ba_0.5_CoO_3−δ_ sample has a slightly curved migration path with *E*_b_ = 2.8 eV. This is expected from steric hindrance; a linear path would encroach on Co (Figures 6a and 7a). Trial calculations using only La^3+^ (Ba^2+^) without relaxing the unit cell yield *E*_b_ = 1.5 eV (3.9 eV) confirming the key importance of steric hindrance. The large Ba^2+^ cation therefore has two contrasting influences in this structure: on the one hand, it expands the lattice, facilitating transport past the smaller La^3+^. On the other hand, its large size leads to a huge energy penalty for ionic migration past Ba^2+^ itself.

Tetragonal LaBaCo_2_O_6−δ_ has a low-energy 2D migration pathway involving the basal plane O2 and apical O3 oxygen sites with *E*_b_ = 1.5 eV (Figures 6b and 7b). 3D transport involves the apical O1 sites in the Ba–O layer and has a much larger *E*_b_ = 3.6 eV (Figure 7b), consistent with the well-established 2D ionic transport in these materials. Direct hopping between O2 sites (within the CoO_2_ plane) has *E*_b_ ∼ 2.2 eV, so it is ∼0.7 eV higher than the favored O2–O3–O2 path. The higher energy of the O2–O2 path is consistent with increased steric hindrance caused by Ba^2+^: at the saddle point, a hopping anion is coordinated to Co^*x*+^ (nearest) and La^3+^ and Ba^2+^ cations (slightly further away). By contrast, the O2–O3 hop involves Co^*x*+^ and two La^3+^ cations, so it is less sterically hindered. The difference between the two paths can be readily seen by inspection of Figure 6b. The importance of steric hindrance from the large Ba^2+^ cation is also evident from a comparison of the calculated *E*_b_ for cubic and layered LaBaCo_2_O_6−δ_. Moving past, La^3+^ has a similar *E*_b_ ∼ 1.5 eV in both structures, while the values for hopping past the large Ba^2+^ cation are substantially larger at *E*_b_ = 3.9 eV (disordered) and *E*_b_ = 3.6 eV (layered). Another point of interest is the similar values of *E*_b_, despite the structures being substantially different.

The vacancy-ordered superstructures have more complex oxygen migration with the orthorhombic symmetry allowing inequivalent 1D channels (Figures 6c–f and 7c,d). The single O3–O2 barrier is split into two paths: a low-energy one along the *a*-direction (the short cell axis) and a higher energy one along the elongated *b*-direction. A further feature is the emergence of local energy minima (labeled i1) next to the vacancy-ordered O3b site (Figure 6c). These occur at (0.5, 0.44, 0.5) and hence are similar to a split atomic site of ∼0.5 Å on either side of the O3b position along the extended *b*-direction. There is only a minimal *E*_b_ ∼ 0.1 eV to move between i1 and O3b sites. The existence of a split site was confirmed by Rietveld analysis with a fitted O3b coordinate (0.5, 0.46, 0.5). The i1 local minimum is, therefore “real” and corresponds to the location of the split O3b site. Additional fits confirmed that there is no splitting along the short *a*-axis, in keeping with the absence of a local minimum in BVSE in that direction. This local minimum is a consequence of the outward displacement of La^3+^ cations, which occurs to maintain coordination with the occupied O3a site (Figure 5 and Table 2). This provides energy stabilization for a split O3b site, moving from a single O3b–La distance (with O3b in the middle between two La^3+^), to a shortened and elongated i1–La distance, with a lower overall energy. The lowest energy 1D path along the short *a*-direction is between O2b–O3b–O2b (*E*_b_ = 1.2 eV), whereas the equivalent path along the *b*-direction involves O3a–O2c–i1–O3b–i1–O2c–O3a (Figure 6c) with three distinct migration barriers. These have calculated values *E*_b_ = 1.5 eV (O3a–O2c), *E*_b_ = 1.4 eV (O2c–i1), and *E*_b_ = 0.1 eV (i1–O3b) as shown in Figure 7c. The highest energy barriers are expected to dominate the ionic transport in this direction. 2D transport thus occurs at an energy above ∼1.5 eV, but, in the first instance, does not involve the O2a site (Figure 6c). A slightly higher energy of ∼1.7 eV is needed to involve all O2 and O3 sites (Figure 7c), and at this point, the transport is equivalent to the tetragonal structure, which has a similar 2D barrier, *E*_b_ = 1.5 eV. The transport through the Ba–O layer continues to carry a high energy penalty with a calculated *E*_b_ = 3.3 eV for migration involving O3a–O2a–O1a (Figure 7c). The equivalent O3b–O2b–O1b path has a slightly higher energy, *E*_b_ = 3.8 eV. Overall, the orthorhombic distortion can therefore be seen to lower the *E*_b_ for some selected migration paths but increase the energy of others.

The orthorhombic distortion in the δ = 0.5 structure is much larger (Table 2 and Figure 4c) and this leads to increased differences between the 1D paths along the a and *b* directions. This is illustrated in Figures 6d–f and 7d. The migration paths have the same basic connectivity, but there are some significant differences, including destabilized O3b, O2b/c, and i1 sites and further reduced *E*_b_ for some pathways. The energies of oxygen ions in some of the crystallographic sites are no longer zero, that is, oxygen ions on the i1, O3b, and O2b sites are destabilized by 0.3–0.4 eV, with the O2c site ∼0.15 eV above zero (Figure 7d). This is caused by the strong distortion of the structure, which leads to unfavored local coordination of the O2b/O3b vacancy sites, but this does not affect structural stability due to the large concentration of vacancies. The O2b–O3b–O2b connection remains the lowest energy path with *E*_b_ = 0.5 eV (∼0.8 eV above zero), which is substantially lower than *E*_b_ = 1.2 eV for the δ = 0.25 structure. The O3a–O2c–i1–O3b–i1–O2c–O3a path along the *b* direction has a rate-limiting highest barrier *E*_b_ = 1.7 eV for the O2c–O3a segment with *E*_b_ = 0.9 eV for the i1–O2c jump, and the i1 split-site again has ∼0.1 eV lower energy than the O3b site (Figure 7d). 2D transport occurs above an energy of ∼1.7 eV, similar to the tetragonal and δ = 0.25 structures (Figure 6e).

A final and significant point is the emergence of a low-energy 3D-connected migration path with *E*_b_ = 2.2 eV (2.4 eV from zero, Figure 7d), hence significantly reduced from *E*_b_ = 3.3 eV for δ = 0.25 and *E*_b_ = 3.6 eV for the tetragonal structure. This involves the O3b–O2b–O1b sites (Figure 6f), with *E*_b_ = 2.2 eV for transport across the Ba–O layer (O2b–O1b), while also benefiting from the low *E*_b_ = 0.5 eV for the O3b–O2b segment. We speculate that the reduced O2b–O1b barrier is linked to the outward motion of the large Ba^2+^ cations due to the large orthorhombic distortion. For the δ = 0.25 structure, the refined Ba-*y* coordinate is 0.252(2) (see Table 2) and is within error at the ideal position observed in the tetragonal structure (*y* = 0.25). For δ = 0.5, the *y*-coordinate is 0.238(2), corresponding to a slight outward displacement of the Ba^2+^ cations (∼0.1 Å), creating more space around the central O1b oxygen site (Figure 6f). The slightly more distant Ba^2+^ cations reduce steric hindrance and facilitate transport through the Ba–O layer *via* the O1b site. The downside is that transport *via* the O1a site is more constricted, and the O2a–O1a path is calculated to have a higher *E*_b_ = 3.9 eV. Nevertheless, ionic transport involving a subset of the oxygen sites is predicted to have a lower effective 3D migration barrier in the δ = 0.5 structure. However, because only a subset is active at this lower energy, the overall ionic diffusivity may be compromised. Furthermore, the increased energies of some of the atomic sites (Figure 7d) suggest that there is additional potential for trapping of oxygen vacancies that would not occur in the basic layered tetragonal structure.

## Discussion

Layered and disordered LaBaCo_2_O_6−δ_ are found to have identical oxygen cycling capacities between 300 and 1000 °C. Rietveld analysis reveals a very similar evolution of the oxygen content/Co oxidation state in these two compositions. The near-identical oxidation state is confirmed both from the refined chemical composition and from bond valence sums using experimental bond distances. This confirms that the oxygen cycling capacity is controlled by thermodynamics (*p*_O_2__) and that the timescale of TGA is slow compared to any kinetic differences between the layered and disordered systems.^2^

BVSE calculations were used to obtain migration barriers for oxide diffusion. These calculations only give barrier energies and do not directly yield ionic diffusion and conductivity, which also depend on connectivity of the migration paths and concentration of mobile ions and vacant sites. Nevertheless, the ease of the BVSE calculations and the fact that they use experimental lattice parameters and atomic coordinates (not relaxed) has helped to provide new insights into the energy barriers that underpin ionic motion. First, the calculations yield similar *E*_b_ in tetragonal-layered and cubic-disordered structures for hopping past A-cations of the same size. Hopping past Ba^2+^ has *E*_b_ ∼ 3.5 eV and past La^3+^ has *E*_b_ ∼ 1.5 eV. This is consistent with MD simulations that give identical *E*_b_ ∼ 0.6 eV for the lowest energy migration path in disordered and layered PrBaCo_2_O_6−δ_.^38,39^ A trial BVSE calculation for layered PrBaCo_2_O_6−δ_ yields *E*_b_ = 0.9 eV (O2–O3), in good agreement with these MD simulations. The higher *E*_b_ = 1.5 eV for LaBaCo_2_O_6−δ_ is consistent with the larger size of La^3+^, leading to increased steric hindrance compared to the smaller Pr ion, and is consistent with established trends in ionic conductivity.^7,8^ In the layered structure, all low-energy paths are located in the central 2D CoO_2_–LnO–CoO_2_ block, enabling an uninterrupted linking of low-energy paths and higher oxide ion diffusivities. In the cubic perovskites, migration paths contain both low- and high-energy barriers, leading to overall lower ionic diffusivities. The BVSE calculations suggest that the low *E*_b_ path is between the O2 and O3 sites. This leads to ionic migration *via* O2–O3–O2 paths, hence involving the LnO and two adjacent CoO_2_ layers. The direct O2–O2 hop in the CoO_2_ planes is not favored as this encroaches on a large Ba^2+^ ion. Experimental *E*_a_ obtained from the high-frequency arc in symmetric cell EIS measurements is ∼1.4 eV for both cubic and layered LaBaCo_2_O_6−δ_.^57,58^ However, this *E*_a_ is known to be affected by porosity and interfacial effects and is not a direct measure of bulk ionic conduction.^59^ For comparison, the much better studied Ln = Pr system has, *E*_a_ ∼ 1.0 eV from symmetric cells^9^ and somewhat lower surface and bulk oxygen diffusion activation energies of 0.7 and 0.5 eV from isotope exchange.^4^ A direct comparison of BVSE migration barriers with ionic conductivity data is therefore not possible. Nevertheless, there does appear to be a good agreement between the BVSE migration barriers and activation energies from EIS on symmetric cells.

The orthorhombic distortion that occurs in the vacancy-ordered δ = 0.25 and δ = 0.5 structures has a strong impact on the ionic migration paths. The reduction in symmetry and elongation of the *b*-axis results in considerable anisotropy: barriers for migration in the *a*-direction are reduced compared to the tetragonal case and enhanced in the *b*-direction. The observed changes in *E*_b_ largely follow the change in lattice metrics (*a* < *b*; Figure 4c) but are also linked to the movement of La^3+^ and Ba^2+^ with a changing internal coordinate, *y* ≠ 0.25. In particular, the emergence of low-energy split-sites (i1) adjacent to O3b and reduction of steric hindrance around the O1b site are linked to displacements of La and Ba cations (Table 2). The calculated *E*_b_ is sensitively dependent on the details of the crystal structure. Relaxing the crystal structure in calculations may therefore significantly affect the accuracy of the theoretical predictions. DFT studies provide the most direct comparison, as DFT and BVSE both use “0 K” structures, whereas the MD simulations proceed *via* calculation of ionic diffusion at high temperatures. To the best of our knowledge, there are only two DFT studies on orthorhombic vacancy-ordered structures in the literature.^32,35^ These both use the idealized vacancy-ordered (δ = 0.5) structure in Figure 1d. The first study on Ln = Pr has lowest *E*_b_ = 0.4 eV for O2b–O2c and *E*_b_ = 0.5 eV for O2b–O3b (using our atom labeling).^32^ This suggests similar barriers for transport within CoO_2_ and between LnO and CoO_2_ planes. A second study on Ln = Gd has lowest *E*_b_ = 1.0 eV (O3b–O2c) and *E*_b_ = 1.2 eV (O2c–O2b), suggesting that the lowest energy migration path runs along the elongated *b*-direction, with the equivalent path along the *a*-direction (O2b–O3b) having a very large 1.8 eV barrier.^35^ This result contrasts with our BVSE results, where transport in the *a*-direction (O2b–O3b) has the lowest energy barrier (Figures 6d and 7c). No atomic coordinates are given in the DFT study on Ln = Gd, so we were not able to directly check this against a BVSE calculation. The reduced barrier in the *a*-direction we observe is consistent with the observed changes in the crystal structure: La^3+^ cations move outward (due to the elongation in the *b*-direction), reducing steric hindrance for transport in the *a*-direction. Despite the well-established nature of these materials, there continues to be uncertainty about the precise link between structure and ionic diffusion, as recently noted in a perspective article.^3^ This is not helped by the large σ_e_ in the LnBaCo_2_O_6−δ_ perovskites that make the direct measurement of the intrinsic ionic conduction using EIS all but impossible.

The emergence of low-energy migration channels in vacancy-ordered structures is of considerable interest for further exploration. These channels (be they 1D, 2D, or 3D) tend to involve a subset of the oxygen sites. However, these are connected *via* (uninterrupted) low-energy barriers, suggesting that they can support improved ionic conduction. This situation is similar to the dimensional reduction that occurs on changing from isotropic cubic to layered tetragonal. In that case, a sixth of the oxygen ions are no longer involved in ionic transport but elimination of Ba^2+^ from the migration path enables substantially improved ionic diffusion in bulk samples. The current work shows that the reduction to orthorhombic symmetry leads to further low-energy migration channels that could lead to improved ionic conductivities. The good agreement between BVSE migration barriers and experimental *E*_a_ from EIS for the tetragonal structures suggests that the low calculated *E*_b_ could indeed correspond to improved ionic conductivities. This is of particular interest for applications at moderate temperatures before the vacancy ordering dissolves (<400 °C). This could include memristors and electrochemical water splitting, where lattice oxygen has been shown to be involved in the reaction.^13^

## Data Availability Statement

Raw data underpinning this article are available through the Heriot-Watt University data repository at https://doi.org/10.17861/3db5d144-6071-4ff6-a934-fc2d7b601f88.

## Supporting Information Available

Photograph of the flow cell used in the neutron diffraction experiment, TGA data, determination of thermal and chemical expansion coefficients, and expanded tables and figures with crystallographic data (PDF)
