# Supplementary material for: Insights into Oxygen Migration in LaBaCo2O6−δ Perovskites from In Situ Neutron Powder Diffraction and Bond Valence Site Energy Calculations
Source: Chem Mater. 2022 Jan 27;34(3):1191–202. doi: 10.1021/acs.chemmater.1c03726 (PMC9007454; doi:10.1021/acs.chemmater.1c03726)
Supplement: Supplementary file 1 — cm1c03726_si_001.pdf [file cm1c03726_si_001.pdf]

Supporting Information for

Insights into oxygen migration in $LaBaCo_2O_{6-\delta}$ perovskites from in-situ neutron powder diffraction and bond valence site energy calculations

Fabian Hesse,[a] Ivan da Silva,[b] Jan-Willem G. Bos[a]*

[a] *Institute of Chemical Sciences, Centre for Advanced Energy Storage and Recovery, School of Engineering and Physical Sciences, Heriot-Watt University, Edinburgh, EH14 4AS, UK*

[b] *ISIS Facility, Rutherford Appleton Laboratory, Harwell Oxford, Didcot OX11 0QX, UK*

* corresponding author: j.w.g.bos@hw.ac.uk

Table S1. Fit parameters used to decouple thermal and chemical expansion, and linear thermal expansion coefficient (α) for $La_{0.5}Ba_{0.5}CoO_{3-\delta}$ and $LaBaCo_2O_{6-\delta}$ (fits shown in Fig. S2).

	$La_{0.5}Ba_{0.5}CoO_{3-\delta}$	$LaBaCo_2O_{6-\delta}$
a_0 (Å)	3.8659	3.8771
b x 10^{-5} (Å K^{-1})	8.49	7.60
c x 10^{-5} (Å K^{-1})	5.33	3.92
T_{red} (K)	541	500
α x 10^{-5} (K^{-1})	2.18	1.95

$$a(T) = a_0 + bT + c(T-T_{red})$$

Table S2. Lattice parameters and unit cell volumes of $La_{0.5}Ba_{0.5}CoO_{3-\delta}$ and $LaBaCo_2O_{6-\delta}$ before and after five heat-cool cycles (RT-1000 °C) under nitrogen determined by XRD.

	$La_{0.5}Ba_{0.5}CoO_{3-\delta}$		$LaBaCo_2O_{6-\delta}$	
	Before	After	Before	After
a (Å)	3.8913(3)	3.8912(3)	3.9183(3)	3.9392(3)
c (Å)			8.0312(5)	7.8255(5)
V (Å^3)	58.92(1)	58.92(1)	123.30(3)	121.43(2)

Table S3. Atomic displacement parameters (Å^2 x 100) for $La_{0.5}Ba_{0.5}CoO_{3-\delta}$ between RT and 1000 °C upon heating and after cooling.

ADP (Å^2 x 100)	RT	250°C	400°C	550°C	700°C	850°C	1000°C	300°C-c	RT°C-c
U(La/Ba/Co)	0.8(1)	1.0(1)	1.4(1)	1.8(2)	2.0(2)	2.7(2)	3.2(3)	1.5(3)	1.3(3)
U_{11}(O)	1.6(2)	1.9(3)	2.1(3)	2.3(3)	3.2(4)	3.9(4)	4.3(5)	2.9(5)	1.8(5)
U_{22}(O)	2.5(1)	2.6(1)	3.6(1)	4.7(2)	4.9(2)	6.5(2)	7.0(3)	4.1(3)	3.1(3)

Free refinement of the ADPs for La/Ba and Co sites gave similar values, so these were constrained to be identical in the final fit.

Table S4. Atomic displacement parameters (Å² x 100) for $LaBaCo_2O_{6-\delta}$ between RT and 1000 °C upon heating and at 350 °C after cooling. Precise determination of ADPs for phase b was not carried out due to the low weight fraction.

ADP (Å² x 100)	RT	250°C	400°C	550°C	700°C	850°C	1000°C	350°C-c
U(La)	0.6(1)	0.7(1)	1.1(1)	1.2(1)	1.8(1)	1.9(1)	2.7(1)	1.4(1)
U(Ba)	0.2(1)	0.3(1)	0.8(1)	0.8(1)	1.2(1)	1.4(1)	1.8(1)	0.5(1)
U(Co1)	0.6(1)	0.7(1)	1.1(2)	1.4(1)	1.7(1)	2.4(1)	2.9(1)	1.9(2)
U(Co2)			1.1(2)					1.5(2)
U_{11}(O1a)	0.7(2)	0.8(3)	1.9(8)	2.0(2)	2.6(2)	3.5(3)	4.5(3)	3.3(8)
U_{22}(O1a)			2.3(9)					3(1)
U_{33}(O1a)	1.4(4)	1.4(4)	8.6(6)	1.8(3)	2.1(3)	2.4(3)	3.5(3)	0.7(6)
U_{11}(O1b)			1.1(8)					4(1)
U_{22}(O1b)			2.2(1)					1.5(9)
U_{33}(O1b)			4.8(8)					2.5(7)
U_{11}(O2a)	2.1(3)	2.5(3)	1.1(5)	3.0(3)	3.9(3)	4.3(3)	7.1(4)	0.7(3)
U_{22}(O2a)	0.9(1)	1.2(2)	3.3(8)	2.0(1)	2.4(1)	3.1(1)	3.5(2)	2.7(8)
U_{33}(O2a)	2.0(2)	2.6(2)	2.6(6)	3.8(1)	5.2(1)	6.1(1)	8.0(2)	2.5(5)
U_{11}(O2b)			1.0(5)					0.9(7)
U_{22}(O2b)			2.0(7)					9(1)
U_{33}(O2b)			2.2(6)					13(2)
U_{11}(O2c)			2.1(3)					4.5(5)
U_{22}(O2c)			1.7(3)					3.9(3)
U_{33}(O2c)			6.0(6)					6.8(5)
U_{23}(O2c)			2.5(3)					3.7(3)
U_{11}(O3a)	2.5(4)	3.5(5)	5(1)	3.9(5)	4.3(5)	4.9(6)	4.8(7)	1.0(5)
U_{22}(O3a)			2.2(9)					2.6(9)
U_{33}(O3a)	1.4(4)	1.8(4)	2.0(5)	2.2(4)	3.3(5)	4.8(7)	8.1(8)	1.8(7)
U_{11}(O3b)			3(2)					21(11)
U_{22}(O3b)			3(2)					15(5)
U_{33}(O3b)			6(2)					16(8)

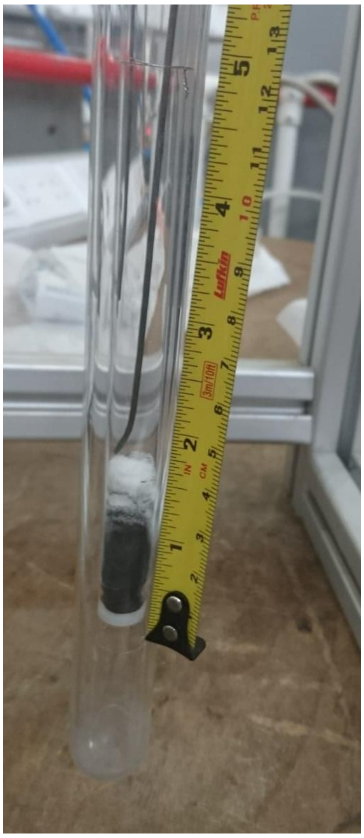

Fig. S1. Photograph of a loaded quartz sample holder for the “gas flow centre stick” vacuum furnace at ISIS. During experiment, 1 bar N_2 is forced to flow through sample ($p_{O2} \approx 10^{-5}$ atm, 100 $cm^3\ min^{-1}$).

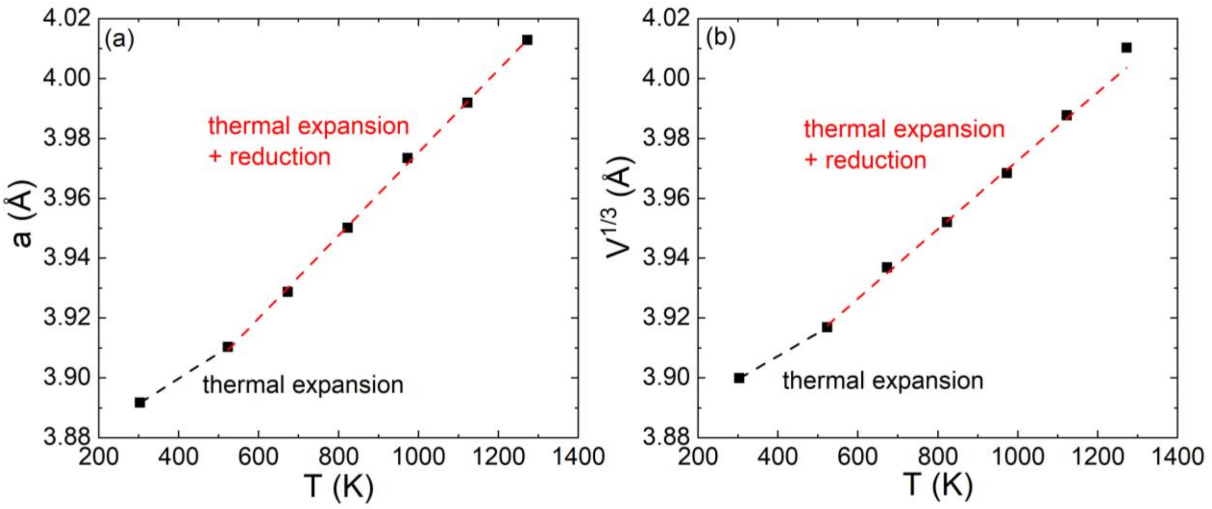

Fig. S2. Temperature dependence of the (pseudo-cubic) lattice parameter for **(a)** cubic $La_{0.5}Ba_{0.5}CoO_{3-\delta}$ and **(b)** layered $LaBaCo_2O_{6-\delta}$ from NPD data. Fit parameters are given in Table S2.

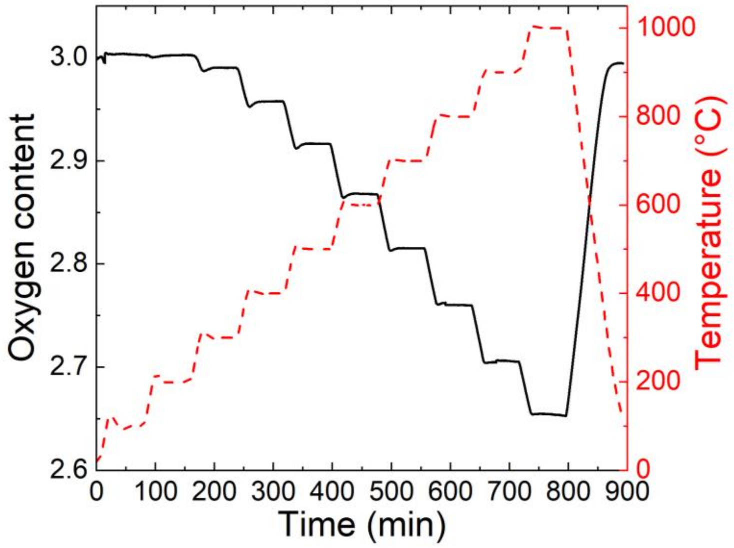

Fig. S3. Stepwise heating and weight profiles of cubic $La_{0.5}Ba_{0.5}CoO_{3-\delta}$ as a function of time, obtained by thermogravimetric measurement under N_2. The temperature was stabilized at 10 temperature steps between RT and 1000 °C for 1h.

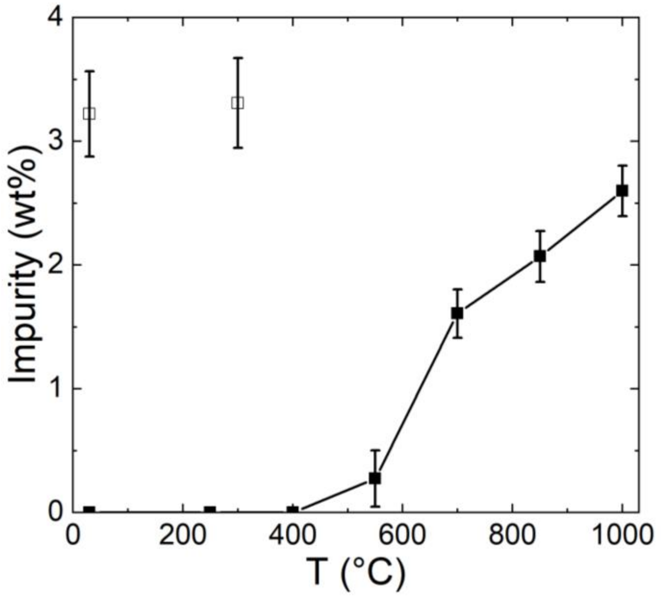

Fig. S4. Temperature dependence of La_2O_3 impurity weight percentage in cubic $La_{0.5}Ba_{0.5}CoO_{3-\delta}$ from Rietveld analysis of NPD data upon heating to 1000 °C (solid squares) and at 300 °C and RT after cooling under N_2 flow (open squares).

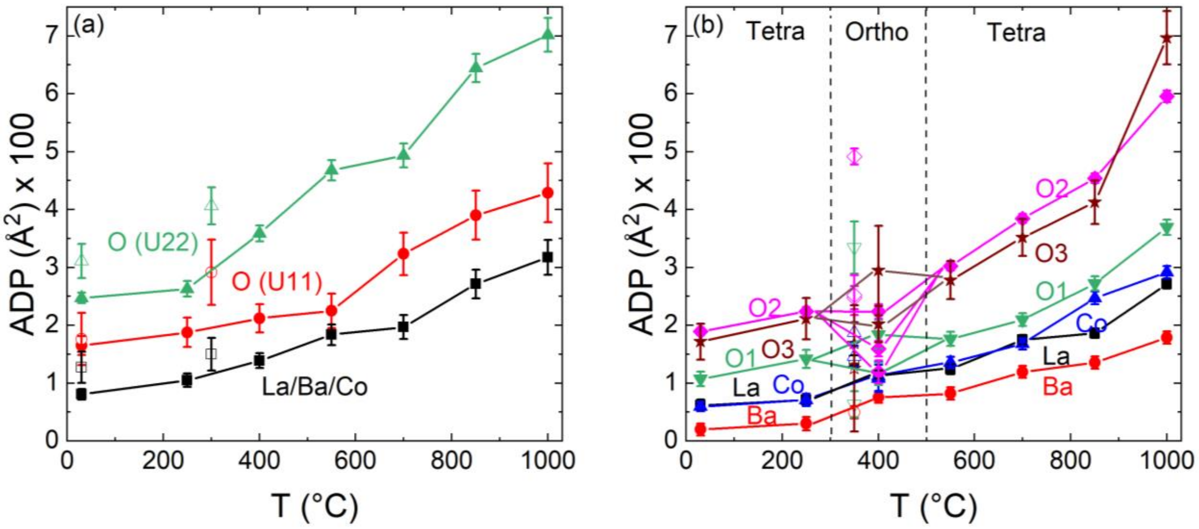

Fig. S5. Temperature dependence of the atomic displacement parameters (ADP) for **(a)** cubic $La_{0.5}Ba_{0.5}CoO_{3-\delta}$ and **(b)** layered $LaBaCo_2O_{6-\delta}$ obtained from Rietveld analysis of NPD data. Open symbols are for data collected on cooling; all datasets collected under flowing N_2.

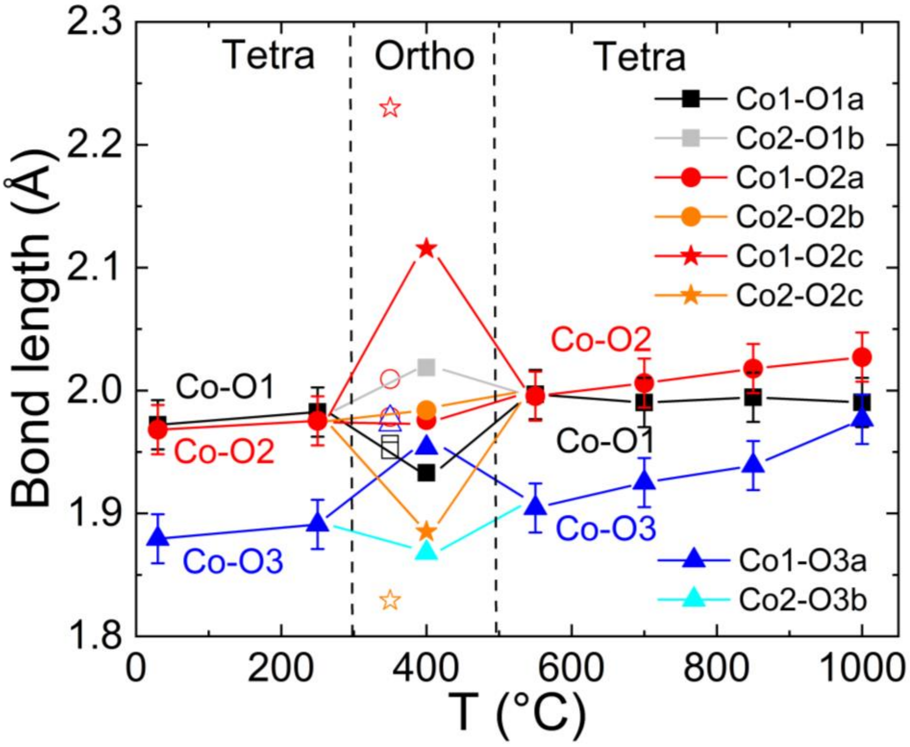

Fig. S6. Temperature dependence of the Co-O bond lengths of $LaBaCo_2O_{6-\delta}$ measured by NPD between RT and 1000 °C upon heating and after cooling (open symbols). Error bars (+/- 0.03 Å) for orthorhombic structures are omitted for clarity.

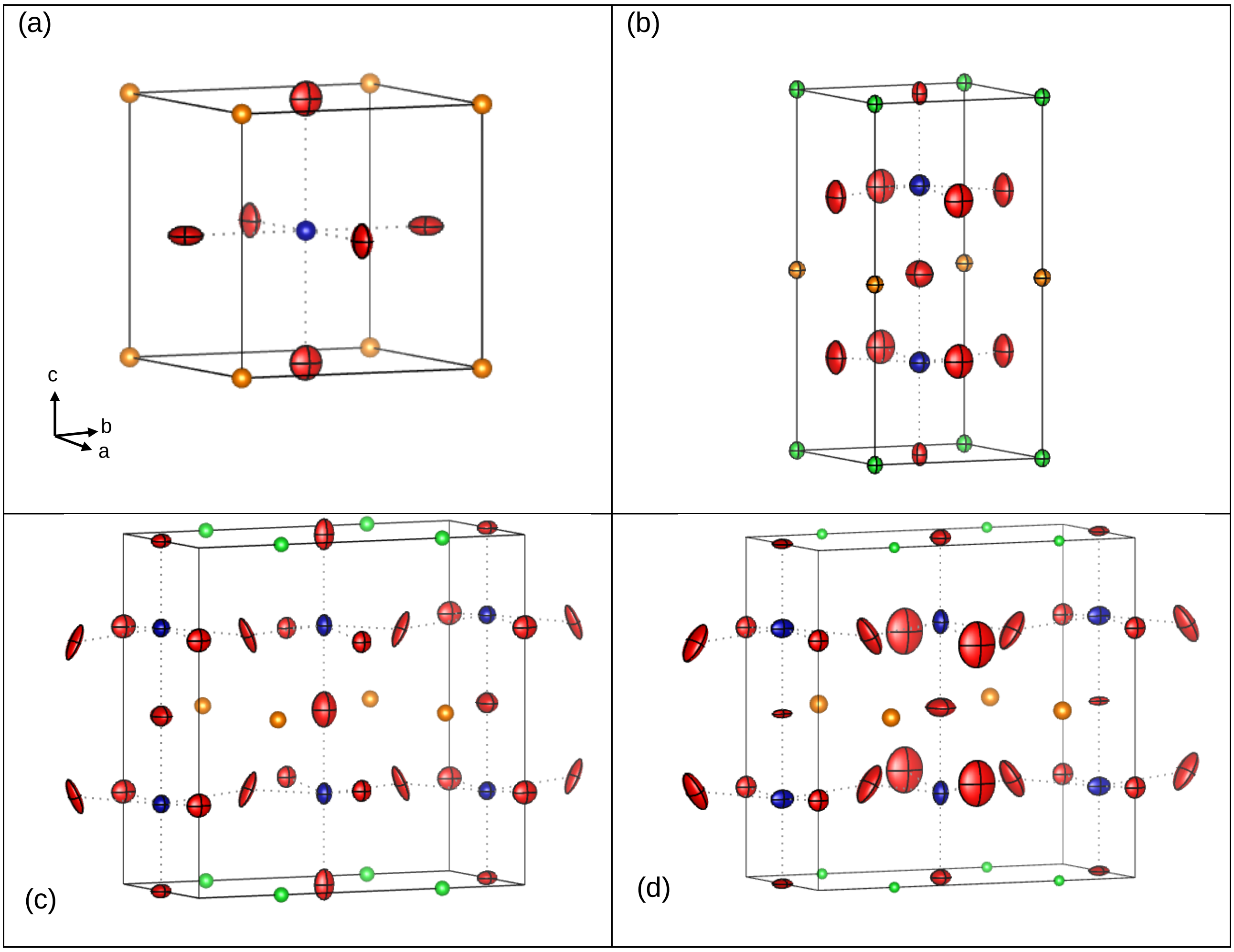

Fig. S7. Schematic representation of anisotropic ADPs for **(a)** cubic $La_{0.5}Ba_{0.5}CoO_{3-\delta}$; **(b)** tetragonal $LaBaCo_2O_{6-\delta}$; **(c)** orthorhombic $LaBaCo_2O_{6-\delta}$ during heating at 400 °C and **(d)** orthorhombic $LaBaCo_2O_{6-\delta}$ during cooling at 350 °C. ADPs are displayed as ellipsoids at 50% probability. La, Ba, Co and O atoms are coloured orange, green, blue, and red, respectively.
